# Evaluation of Perception, Awareness, and Practices Related to Burns First Aid: Largest Cross-Sectional Study Among Non-Healthcare Providers in the Western Region of Saudi Arabia

**DOI:** 10.7759/cureus.33839

**Published:** 2023-01-16

**Authors:** Rakan Abu alqam, Basim Awan, Badr Alsulymani, Louae Malaika, Mohammed Al-Rezqi, Abdulrahman A Malaikah, Saad H Alqarni

**Affiliations:** 1 Faculty of Medicine, King Abdulaziz University, Jeddah, SAU; 2 Division of Plastic Surgery, Department of Surgery, Faculty of Medicine, King Abdulaziz University, Jeddah, SAU

**Keywords:** jeddah, burns first aid, practices, perception, non-healthcare providers

## Abstract

Introduction

Burn injuries are among the most catastrophic public health issues because of the severe physical, functional, and psychological effects. Numerous studies have revealed that both developed and developing societies lack understanding about first aid for burns. This research sought to review and appraise perception, awareness, and practices of burn first aid among non-healthcare providers in Jeddah, Saudi Arabia, and whether they need an effective program. To the best of our knowledge, this is likely the first research conducted in Jeddah.

Methodology

We conducted a cross-sectional study in Jeddah, Saudi Arabia using a self-administered online questionnaire among non-healthcare providers in July 2022. The questionnaire was made up of 29 questions divided into two sections: demographics and first aid for burns. The Unit of Biomedical Ethics Research Committee at the Faculty of Medicine, King Abdulaziz University, Jeddah, Saudi Arabia approved this study.

Results

This study included 575 participants. Males comprised 54.8% (315) and females 45.2% (260) of all respondents. A total of 443 respondents (77%) held a university diploma. All respondents had a mean burn knowledge score of 6.35±1.43 out of eight. Traditional medication was used on the burn by 484 people (84.2%). Antibiotic use in burn injuries was poorly understood as 453 (78.8%) of study participants agreed that antibiotics are beneficial in the case of burns, which is incorrect.

Conclusion

The level of first-aid practices for burn patients among non-healthcare workers was insufficient, and the use of traditional medicines and antibiotics in burn patients was excessive. The findings of this study should be carefully considered by various healthcare organizations.

## Introduction

Burn-related injuries occur when one or more of the skin's layers ruptures by hot liquids, hot solids, or a flame. Additionally, 'burn' is a term used to describe damage to the skin brought on by radiation, electricity, or chemicals [1]. Burn injuries are among the most expensive and catastrophic public-health issues because of the severe physical, functional, and psychological effects [2,3]. It might result in prolonged hospital stays, deformity, and incapacity [4]. Many burn victims experience mental health issues, particularly depression and post-traumatic stress disorder [5]. Burns have more severe negative consequences on immunocompromised people [6].

These injuries rank as the fourth most frequent type of trauma worldwide [7]. They are one of the most common forms of injuries globally, causing 11 million injuries and 265,000 deaths each year [8]. The prevalence of burn injuries in Saudi Arabia ranged from 112 to 518 per 100,000 per year, with households accounting for 72-94% of incidents [9]. According to WHO, more than 95% of these burn injuries occur in developing countries [10]. Previous research stated that the rate of child burn deaths is seven times higher in low- and middle-income countries than in higher-income countries [6,11]. 

The primary goal of first aid in burns is to limit tissue injury, stabilize the vasculature, minimize edema, give the right level of analgesia, and improve outcomes [12]. So improving the first-aid knowledge and steps of primary burn prevention is essential to each individual [13]. First aid is urgent care given immediately for patients to keep their functional organs or save a patient's life [14]. It effectively decreases the chance of prolonged hospitalization and provides plenteous finance, especially in improving advanced countries [15].

Optimal first aid includes "stripping clothes, applying cold water for 20 minutes, organizing help, and putting on the sterile dressing (STOP)" [12]. Within three hours, nice cold tap water should be administered after the burn injury, and the temperature should be between 12-18°C [12]. Without any conclusive scientific proof, individuals have been utilizing a variety of topical treatments for burn injuries for many years and are still doing so today [16]. Ice, herbal medicines, oil, honey, vinegar, flour, toothpaste, eggs, and even a mixture of urine and mud are among the most often utilized materials [2,12]. Giving such odd remedies worsens the wound and encourages bacterial development, increasing the post-burn consequences [16]. According to studies, using ice to treat big surface area burns, particularly on young children and elderly patients, is largely related to an increase in tissue damage and an increased risk of hypothermia [14,17].

This study is limited by the sample size, which comprises the individuals who participated after they were reached out to with the questionnaire. Furthermore, using a multiple-choice format to test the sample's understanding of burn first aid may have overestimated the participants' knowledge. Some participants may have given correct answers by chance. In contrast, others may have answered correctly, but they might not have been able to perform those actions when faced with a real-world first-aid scenario. Research has been conducted worldwide to demonstrate how well-developed and developing nations understand first aid for burns. However, most of these studies reveal a need for more proficiency in the non-medical sector. Research findings concerning Saudi Arabia's burn-related first-aid knowledge are generally consistent with those from other societies. However, few targeted the general population, and virtually no studies were conducted in Jeddah, Saudi Arabia, regarding first-aid burn perception and awareness. This study intends to assess how non-healthcare practitioners in Jeddah, Saudi Arabia perceive and are aware of burn injury first-aid. Hopefully, this study would encourage additional research and persuade legislators to develop improved burn-injury care recommendations.

## Materials and methods

Study design and data collection

This observational cross-sectional study was formulated via an online Google Form questionnaire during July 2022. This self-administered survey was sent randomly to people living in Jeddah, Saudi Arabia via Instagram, Twitter, WhatsApp, and other social media platforms to assess the knowledge and awareness of burn's first aid among non-healthcare providers. All participants were informed about the prerequisites of the study. 

Questionnaire variables

The questionnaire was formulated based on our study's objectives and available questionnaire with similar objectives [1]. A language translation specialist translated the questionnaire into Arabic. The questionnaire had 29 questions divided into two sections: demographics and questions about burn injury and first aid. The first section asked for age, gender, nationality, job position, marital status, and whether or not the respondent has children in the household. The second section aimed to assess the knowledge about first aid for burns and the use of any kind of traditional remedies such as coffee, honey, aloe vera, or toothpaste. Our research evaluated knowledge and practices out of 16: the overall score for both knowledge and practices ranged from 1 to 14; a higher score indicated more awareness about first aid for burns.

Statistical analysis

The baseline characteristics of the respondents and correct answers to questions about burns first-aid knowledge and practices were presented using descriptive statistics. The mean burn knowledge and practice scores were calculated by counting the number of correct responses to each question per respondent. The association between the mean burn knowledge and practices score and age, marital status, sex, job position, nationality, burn training course participation, and the experience of a burn injury to oneself or a family member were assessed using the descriptive one-way Analysis of Variance (ANOVA) test. Statistical Package for Social Science (SPSS) version 21.0 was used for the analysis, which produced 95% confidence intervals.

Ethical consideration

Ethical approval was obtained from the Unit of Biomedical Ethics Research Committee at the Faculty of Medicine, King Abdulaziz University, Jeddah, Saudi Arabia with the reference number 175-22. All participants agreed after being informed of the study's goals and the anonymity of their responses, and online consent was obtained from all participants.

## Results

A total of 575 respondents were included in this study. More than half of the respondents, that is, 315 (54.8%), are male. Most of the respondents (27.1%) were aged between 22 and 29, and the majority (91%) were Saudi Arabian. More than two-thirds, that is, 443 (77%), were university graduates and most (185 (32.2%)) were students. The vast majority (60%) had a monthly income of <10,000 SAR. A considerable percentage of the respondents (343 (59.7%)) had suffered burn damage to themselves or their families. Yet, only 130 (22.6%) had taken a burn training course. The majority of respondents (416 (72.3%)) lived with children, adolescents, or teenagers under 18 years. Many (381 (66.3%)) do not have fire extinguishers at home (Table 1).

**Table 1:** Baseline characteristics of the respondents

Characteristic	n	Percentage
Gender		
Female	260	45.2
Male	315	54.8
Age		
18-21	103	17.9
22-29	156	27.1
30-39	86	15
40-50	93	16.2
>50	137	23.8
Nationality		
Non-Saudi	52	9
Saudi	523	91
Educational level		
Primary	4	0.7
Intermediate	11	1.9
High school	117	20.3
University	443	77
Employment		
Student	185	32.2
Government sector employee	154	26.8
Privet sector employee	104	18.1
Self-employed	38	6.6
Unemployed	94	16.3
Monthly income		
<10,000 SAR	345	60
10,000-20,000 SAR	150	26.1
21,000-30,000 SAR	53	9.2
>30,000 SAR	27	4.7
Marital status		
Single	267	46.4
Married	272	47.3
Divorced	27	4.7
widowed	9	1.6
With children/adolescent/teenagers (under 18 years) living at home		
Yes	416	72.3
No	159	27.7
Participated in burn training course		
Yes	130	22.6
No	445	77.4
Do you have a first-degree relative in medical field?		
Yes	276	48
No	299	52
Do you have a fire extinguisher at home?		
Yes	194	33.7
No	381	66.3
Have you ever experienced a burn injury before to yourself or family?		
Yes	343	59.7
No	232	40.3

**Figure 1:**
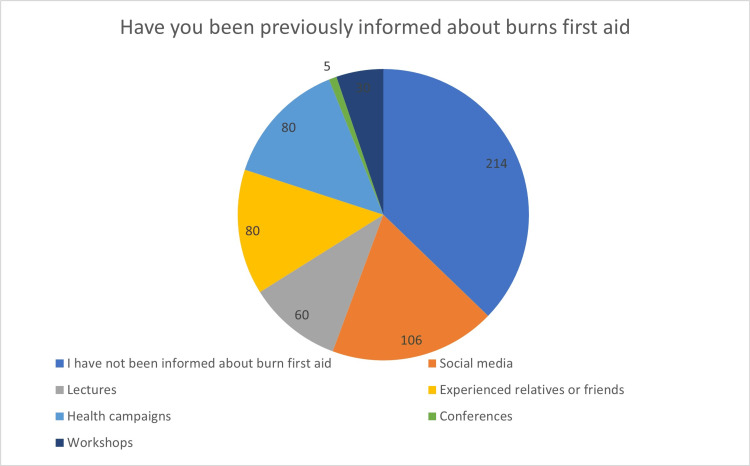
Respondents’ source of information regarding first aid for burns

Of 575, 214 (37.2%) had not been informed about burn first aid, and the least common source of information was “conferences”, with only five (0.9%) of the respondents (Figure 1).

**Figure 2:**
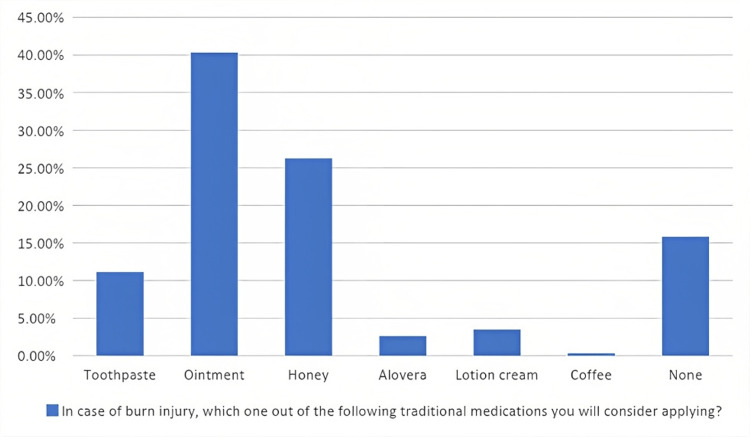
Traditional remedies for burned injured areas

Table 2 shows the assessment of respondents' knowledge and practices in dealing with burns. The knowledge and practices of participants in dealing with burns are presented Using eight questions & statements for knowledge and eight for practices, of all responders, 253 (44%) knew that washing the burned area with cool water was the first correct step for treating a burn trauma. Additionally, 453 (78.8%) participants concurred that antibiotics are beneficial in the case of burns. Only 72 (12.5%) individuals knew that they should administer water to a burn injury for 10 to 15 or >15 minutes. Approximately 138 (24%) of the respondents used room temperature water between 20 and 25°C. The respondents' knowledge of electrical burns appeared to be improving, as 514 (89.4%) of them knew that the first step in treating an electrical burn is turning off the source of electricity (Table 2). Nonetheless, 484 responders (84.2%) opted to treat the burn area using traditional remedies (Figure 2).

**Table 2:** Knowledge and practices of respondents on burn first aid

Knowledge statement	Correct answer	True N(%)	False N(%)
Burn can lead to permanent injuries.	Agree	500 (87)	75 (13)
Burn injuries can lead to mental disorders.	Agree	506 (88)	69 (12)
In case of burn injury, covering the burned area before heading to the hospital can decrease the risk of infection.	Agree	305 (53)	270 (47)
In case of burn injury, picking blisters is an incorrect action.	Agree	306 (53.2)	269 (46.8)
Applying first-aid medicine at home over a burned area leads to a better outcome.	Agree	452 (78.6)	123 (21.4)
In case of electrical burn injury, I should not touch the injured person if he/she is still in contact with electrical current.	Agree	514 (89.4)	61 (10.6)
In case of electrical burn injury, first action is to turn off the source of electricity if possible.	Agree	517 (89.9)	58 (10.1)
If we know that young children are more susceptible to domestic burns, do you take certain measures to prevent this from happening, such as not letting children play around the tea or coffee table or in the kitchen while cooking?	Yes	552 (96)	23 (4)
Practices statement	Correct answer	True N(%)	False N(%)
Washing the burned area with room temperature water is the first correct step in case of burn injuries.	Agree	253 (44)	322 (56)
In case of burn injury, which one out of the following traditional medications you will consider applying?	None	91 (15.8)	484 (84.2)
How to extinguish a pot of oil caught on fire	Cover with clothes	467 (81.2)	108 (18.8)
In case of burn injury, apply water for	10-15 minutes, >15 minutes	72 (12.5)	503 (87.5)
The best way to expose a large burn area to moderate water temperature is by	Keeping it under the shower water	223 (38.8)	352 (61.2)
If you know that the temperature of cold water from the refrigerator is 4-6°C, what is the preferred temperature of water to be used for burn-injury first aid?	Room temperature water 20-25°C	138 (24)	437 (76)
In case of burn injury, if your clothes were caught in fire you should roll on ground.	Agree	449 (78.1)	126 (21.9)
In case of burn injury, it is beneficial to use antibiotics in its management.	Disagree	122 (21.2)	453 (78.8)

Table 3 shows the results of previous studies done to document the knowledge of implementing first-aid steps among the population. In our study, results were that 24% of the respondents applied room temperature water (20-25°C), and only 5.6% applied the water for an optimal 20 minutes or more. The prevalence of burn first-aid management knowledge and practices is measured. Based on the results, the mean knowledge score was 6.35 (SD 1.43) out of eight points. The majority, 303 (52.7%), had an excellent level of knowledge. For practices, the mean score was 3.15 (SD 1.42) out of eight points. Only 11 (1.9%) had an excellent level of practice (Table 4).

**Table 3:** Burns first-aid practices literature summary

Author	Location	Burns first-aid practices
Harvey et al. [6]	Sydney, Australia	82% of respondents applied water to cool burn wounds 9.4% applied the water for optimal 20 minutes
Cuttle et al. [24]	Australia	80.2% of respondents applied cold water to cool burn wounds 12.1% applied the water for an optimal 20 minutes or more
Karaoz B et al. [25]	Milas, Turkey	39.6% of respondents applied cold water to cool burn wounds
Scheven D et al. [26]	KwaZulu-Natal, South Africa	26% of respondents applied cold water to cool burn wounds

**Table 4:** Prevalence of knowledge and practices related to burn first aid

Statement	N (%)
Knowledge score (mean ±SD)	6.35±1.43
Level of knowledge	
Poor	13 (2.3)
Acceptable	39 (6.8)
Good	220 (38.3)
Excellent	303 (52.7)
Practices score (mean ±SD)	3.15±1.42
Level of practices	
Poor	191 (33.2)
Acceptable	289 (50.3)
Good	84 (14.6)
Excellent	11 (1.9)

Measured variations in knowledge and behaviors are caused by participant socio-demographic traits, prior burn injury history, and first-aid management. The oldest age group (>50 years) was shown to have considerably higher knowledge scores (P = 0.000). Likewise, those with a high monthly income of >21,000 SAR got a high practice score (P = 0.000). Additionally, those who took a burn training course scored better on the practice test (p = 0.000) (Table 5).

**Table 5:** Statistical mean differences of knowledge and practices in relation to the socio-demographic characteristics, previous history of burn injury, and participation in burn training course

Factor	Knowledge score (8) Mean ± SD	P value	Practices score (8) Mean ± SD	P value
Gender				
Female	6.41±1.39	0.330	3.14±1.39	0.874
Male	6.29±1.46		3.16±1.45	
Age group				
18-21	6.24±1.47	0.000	3.00±1.50	0.708
22-29	6.05±1.61		3.12±1.34	
30-39	6.51±1.22		3.18±1.49	
40-50	6.16±1.49		3.24±1.53	
>50	6.79±1.11		3.23±1.32	
Nationality				
Non-Saudi	6.17±1.45	0.347	3.25±1.41	0.620
Saudi	6.36±1.43		3.14±1.42	
Educational level				
Primary	8.00±0.00	0.071	3.25±0.95	0.427
Intermediate	5.81±2.22		2.45±0.82	
High school	6.40±1.30		3.14±1.39	
University	6.33±1.43		3.17±1.44	
Employment				
Student	6.21±.56	0.136	3.10±1.49	0.965
Government sector employee	6.60±1.27		3.18±1.47	
Privet sector employee	6.25±1.42		3.22±1.45	
Self/ employed	6.36±1.51		3.10±1.08	
Unemployed	6.29±1.36		3.17±1.27	
Monthly income				
<10,000 SAR	6.28±1.49	0.516	3.03±1.39,	0.000
10,000-20,000 SAR	6.38±1.38		3.10±1.40	
21,000-30,000 SAR	6.56±1.02		3.81±1.45	
>30,000 SAR	6.51±1.50		3.74±1.45	
Marital status				
Single	6.21±1.51	0.087	3.10±1.48	0.780
Married	6.43±1.36		3.18±1.38	
Divorced	6.74±1.22		3.33±1.17	
Widowed	6.88±1.16		3.33±1.22	
With children/adolescent/teenagers (under 18 years) living at home				
Yes	6.31±1.48	0.294	3.12±1.40	0.428
No	6.45±1.27		3.23±1.46	
Participated in burn training course				
Yes	6.44±1.60	0.391	3.68±1.61	0.000
No	6.32±1.37		3.00±1.32	
Do you have a first-degree relative in medical field?				
Yes	6.40±1.45	0.350	3.20±1.49	0.453
No	6.29±1.41		3.11±1.35	
Do you have a fire extinguisher at home?				
Yes	6.44±1.50	0.246	3.25±1.43	0.224
No	6.30±1.39		3.10±1.41	
Have you ever experienced a burn injury before to self or family?				
Yes	6.41±1.31	0.202	3.21±1.51	0.203
No	6.25±1.59		3.06±1.27	

**Figure 3:**
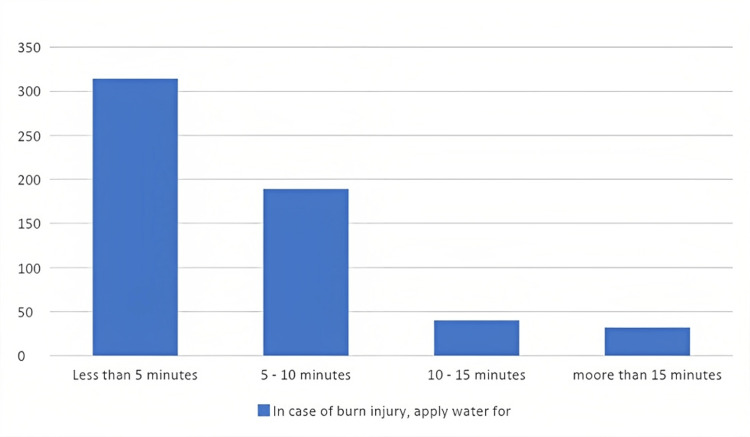
In case of burn injury, apply water for how many minutes ?

Lastly, Figure 3 shows that most participants chose to apply water to the burned area for less than five minutes.

## Discussion

This study sought to investigate and evaluate the burn first-aid knowledge, attitudes, and practices among non-healthcare providers in Jeddah, Saudi Arabia, and determine whether the general public requires an efficient program. 575 respondents were included in this study. 54.8% (315) of the responders were men. Most participants were aged between 22 and 29 (27.1%). 

Interestingly, 484 people (84.2%) used conventional medicine for the burn area. The study's 453 (78.8%) participants who agreed that antibiotics help treat burns had a poor understanding of the use of antibiotics in burn injuries. Strangely, only 253 (44%) knew that applying cold water to a burned area was the proper initial procedure. Like two previous studies in Saudi Arabia, social media was our study population's most popular source of information, while conferences were the least popular [7,13]. This could be due to the accessibility of social media apps to people of different ages. In contrast, two more studies in New South Wales, Australia, and Saudi Arabia revealed that official programs and firs-aid manuals were the most often cited sources of burn first-aid knowledge [2,18]. The only explanation we found is that it depends on the geography of the community; different states, provinces, cities, or neighbors have different interests, some of which could be social, medical, or cultural.

Honey is believed to have the ability to stimulate tissue growth, increase epithelialization, and reduce scar formation, making it a potentially promising therapy [19]. As seen in Figure 2, more than 25% of participants choose to use honey out of traditional remedies as a first-aid treatment for burns. Research conducted in Saudi Arabia revealed that 69.9% of respondents treated their burns using honey [2]. It should be noted that current findings from a systematic review comparing the use of honey to other traditional dressing procedures revealed major uncertainties and may delay the healing capability of partial and full-thickness burns when compared to early excision and grafting [20,21]. Solutions like honey might be related to financial factors: With low monthly income, cheaper solution might be used.

All respondents' average knowledge score for burn first aid was 6.35 ± 1.43 out of eight. However, the mean burn first-aid practices were 3.15 ± 1.42 out of eight. Only 11 people (1.9%) in our study were found to have a remarkable degree of practice awareness and to be fully aware of the given burn situations (Table 4). On the other hand, more than half of the respondents had insufficient information about appropriately handling burn patients during first aid, preventing them from running the situation. There was a general dearth of understanding regarding burn first-aid practices in Riyadh, Al Majma'ah, and Al-Ahsa [2,7,22]. This underlines the need to hold campaigns and training sessions for burn avoidance and appropriate first-aid administration. Additionally, knowledge ratings were much higher in those who received and practiced burn first-aid care. This is in line with the findings of an earlier investigation [16]. Again, it demonstrates the need to provide courses to the general public on effective first-aid administration with burns. 

Contrary to other research in the literature, our study did not find a significant correlation between higher education level and outstanding first-aid management [2,22,23]. Our study's findings revealed that just 5.6% of respondents administered water for the recommended 20 minutes or more, while 24% used room temperature (20-25°C) water. Harvey et al. conducted a telephone poll of 7,320 people in New South Wales, Australia, and found that 82% of respondents said they would use water to cool a burn, but only 9.4% would do so for the recommended 20 minutes [6]. Less than 1% of respondents said they would use additional first-aid techniques [6]. In Australia, Cuttle et al. evaluated 459 burn victims retrospectively [24]. Of them, 80.2% used cold water, although only 12.1% did so for 20 minutes or longer [24]. 39.6% of burn victims in Milas, Turkey, treated their wounds with only cold water [25]. While 26% of people in Kwa-Zulu Natal applied water, just 1% applied it for 10 minutes [26]. This can be connected to the socioeconomics of the various nations and their various educational programs.

It is essential to be aware of this study's limitations. There may be a potential for selection bias for several questions due to the small sample size. Similar to most cross-sectional studies, a correlation was discovered rather than a cause for this lack of knowledge and behaviors. However, this study might serve as a starting point for future research.

## Conclusions

The non-healthcare providers in Jeddah, Saudi Arabia, are said to be well knowledgeable about first aid. This wealth of information is greatly influenced by social media, friends, and family. The people of Jeddah has been found to be relatively unaware about first-aid practices, despite the fact that the vast majority of survey participants (77%) were well educated and held bachelor's degrees. Moreover, the results of this study confirmed the need for an effective educational program for non-healthcare providers to enhance their knowledge and practices regarding first aid for burns, which will indirectly increase the success and provision of ideal treatment for the patients. 
